# Study on method to achieve high transmutation of LLFP using fast reactor

**DOI:** 10.1038/s41598-019-55489-w

**Published:** 2019-12-16

**Authors:** Toshio Wakabayashi, Yoshiaki Tachi, Makoto Takahashi, Satoshi Chiba, Naoyuki Takaki

**Affiliations:** 1 Tohoku University, Graduate School of Engineering, 6–6–11 Aoba, Aramaki, Aoba-ku, Sendai, Miyagi 980–8579 Japan; 2 Oarai Research and Development Institute, Japan Atomic Energy Agency, 4002, Narita-cho, Oaraimachi, Ibaraki 311–1393 Japan; 3 Laboratory for Advanced Nuclear Energy, Tokyo Institute of Technology, 2–12–1 Ookayama, Meguro-ku, Tokyo, 152–8550 Japan; 4 Department of Nuclear Safety Engineering, Tokyo City University, 1–28–1 Tamazutsumi, Setagaya-ku, Tokyo, 158–8557 Japan

**Keywords:** Energy science and technology, Nuclear energy, Materials science, Structural materials, Composites

## Abstract

The purpose of this study is to clarify the method to achieve high transmutation rates of four long-lived fission products (^79^Se, ^99^Tc, ^107^Pd, and ^129^I) using a fast reactor. New LLFP target assemblies were invented in consideration of the suppression of thermal spikes in adjacent fuel assemblies by combining YD_2_ and YH_2_ moderators or using a thermal neutron filter material. It was clarified that the high transmutation rate of about 8%/year was achieved, if the new LLFP target assemblies of 4 nuclides were loaded in the blanket region of the sodium cooled, MOX fueled fast reactor. The feasibility of the LLFP transmutation target was clarified through experiments on material properties and fabrication of the LLFP target, YH_2_ and YD_2_ moderators.

## Introduction

The fast reactor can be used for various purposes such as breeding, the transmutation of long-lived fission product(LLFP) and minor actinide(MA) using surplus neutrons. Many studies have been performed on the transmutation of MA and LLFP^1–26^. With regard to LLFP, a transmutation study on six nuclides, which are important from the viewpoint of reducing environmental impact, has been conducted^27^. The six LLFPs and their half-lives are ^79^Se: 327,000 years, ^93^Zr: 1,570,000 years, ^99^Tc: 211,000 years, ^107^Pd: 6.5 million years, ^129^I: 15.7 million years, and ^135^Cs: 2.3 million years. As a result, it was shown that using a fast reactor, transmutation exceeding the support ratio(SR) 1 for each of these six nuclides can be achieved using YD_2_ moderator^27^. The support ratio is defined as the ratio of the amount of transmuted LLFPs to the amount of LLFPs produced in the core fuel over the same period of time in a reactor. In this case, the elements of LLFP are used without isotopic separation of the six nuclides. We also clarified a method of transmuting six nuclides simultaneously in one fast reactor^28^. However, in these methods, the transmutation rate of LLFP nuclides was low, and it was necessary to load a large amount of LLFP and to recycle a large amount of LLFP targets. This causes a significant loss of LLFP during recycling. Achieving a high transmutation rate also leads to a reduction in loss when recycling LLFP targets. It is difficult to achieve a high transmutation rate for all six nuclides. In particular, ^135^Cs and ^93^Zr are considered to be very difficult due to their low neutron absorption cross sections. It can be expected that the PUREX process at the reprocessing can separate 98% of Tc, 99% of I, 99.5% of Pd, and 90% of Se^9,29,30^. Therefore, it is considered that the influence of other FPs and impurities on the transmutation characteristics is small.

^79^Se, ^99^Tc, ^107^Pd, and ^129^I were selected as nuclides aiming at a high transmutation rate. The reason is as follows:

LLFP is considered important from the viewpoint of the safety performance of the repository (exposure dose to the public in the far future)^31^. Among the 4 nuclides, it has been pointed out that the nuclide that poses a problem in the long-term radiological effects in geological disposal is ^129^I, which is a long-lived nuclide that is soluble and has low absorption to the underground material. ^99^Tc is the dominant radioactivity of the vitrified radioactive waste, and its potential toxicity is an issue. ^79^Se has also become a determinant of public exposure in the period of 10^4^–10^5^ years. ^107^Pd is important in terms of overall exposure reduction.

In order to achieve a high transmutation rate, it is necessary to increase the number of thermal neutrons by increasing the ratio of moderator in the LLFP target with moderator. For this purpose, in addition to deuterium D, which has been used as a moderator to the present^32–34^, it will be necessary to apply hydrogen. We consider achieving a high nuclear transmutation rate by combining deuterium and hydrogen. In addition, when hydrogen is used, it is anticipated that thermal neutrons and the power density increase (thermal spike) at the fuel pins in the fuel assembly adjacent to the LLFP target assembly, and countermeasures are also required.

The purpose of this study is to clarify the method to achieve high transmutation rates of four long-lived fission products (^79^Se, ^99^Tc, ^107^Pd and ^129^I) using a fast reactor. Another purpose of this study is to clarify the feasibility of the LLFP transmutation target by experiments on material properties and fabrication.

## Results and Discussions

### Study on high transmutation performance of ^79^Se, ^99^Tc, ^107^Pd and ^129^I

**Figure 1:**
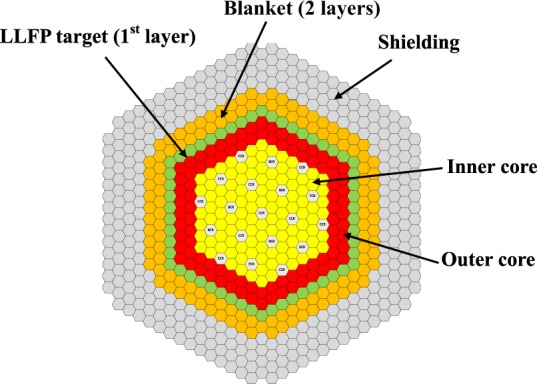
Core configuration of LLFP loaded core of 300 MWe class sodium cooled, MOX fueled fast reactor. The core has two homogeneous zones: inner and outer cores. The LLFP target assemblies are loaded in the first layer of the blanket region. The blanket fuel assemblies are loaded in the second and third layers of the blanket region.

**Figure 2:**
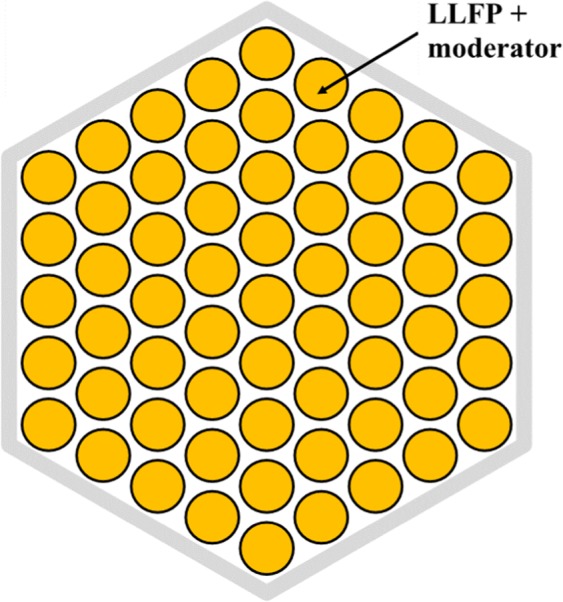
Arrangement of LLFP target pins in LLFP target assembly. There are 61 LLFP target pins in the LLFP assembly. Each LLFP target pin is composed of LLFP and moderators. YD_2_ and YH_2_ are used as moderator.

**Figure 3:**
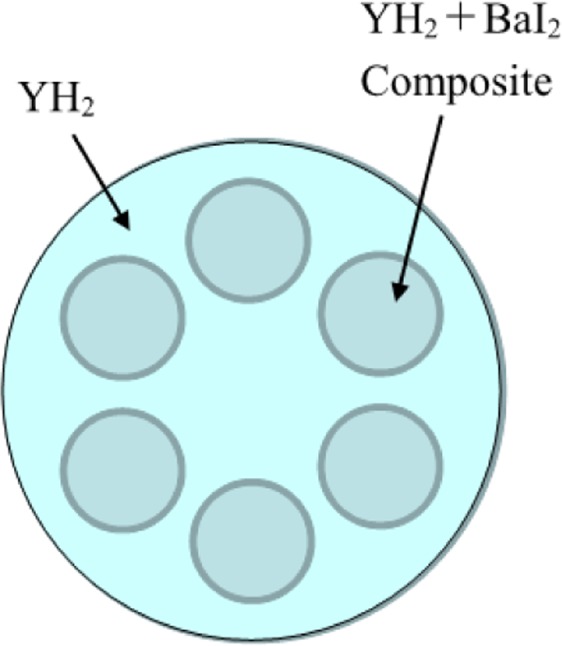
Example of Arrangement of LLFP and moderator in LLFP target pin. LLFP target of ^129^I or ^79^Se is a complex in which BaI_2_ or ZnSe compound is inserted into the holes provided in a moderator pellet.

Table 1 shows the main core specifications of the 300 MWe class sodium cooled, MOX fueled fast reactor. The isotopic composition of the four nuclides is shown in Table 2. Figure 1 shows the core configuration of LLFP loaded core. Figure 2 shows an arrangement of LLFP target pins in LLFP target assembly. Figure 3 shows an example of the arrangement of LLFP and moderator in LLFP target pin.

**Table 1:** Specifications of fast reactor core.

Items	Units	Specifications
Reactor thermal power	MWt	710
Core type		Two-zone homogeneous
Core diameter	mm	1800
Core height	mm	930
Axial blanket thickness (upper/under)	mm	300/350
Operation cycle length	days	123
Residence time of ordinary fuel	days	492
Fuel type		MOX
Heavy metal (ton) (inner/outer/blanket/total)		3.5/3.0/26/32.5
Core fuel assemblies (Inner/Outer/Total)		108/90/198
Pu enrichment (Inner/Outer)	%	22.8/30.1
Pu isotopic composition* ^238^Pu/^239^Pu/^240^Pu/^241^Pu/^242^Pu		1.0/65.9/25.2/3.9/4.0
Control rods		19
Average total flux (inner/outer/LLFP/blanket)	10^15^/cm^2^ sec	4.1/2.4/1.2/0.5
Ratio of fast neutron flux to total neutron flux in the core		0.6
Power share of blanket Axial/radial	%	2.8/1.7

*Pu isotopic composition is same in each region.

**Table 2:** Isotope abundance of loaded LLFP nuclides.

Isotopes of loaded LLFP elements	Abundance (%)
^76^Se	0.027
^77^Se	2.786
^78^Se	5.587
^79^Se	13.32
^80^Se	22.75
^82^Se	55.52
^99^Tc	100.00
^104^Pd	2.93
^105^Pd	35.14
^106^Pd	17.86
^107^Pd	21.72
^108^Pd	17.12
^110^Pd	5.24
^127^I	23.91
^129^I	76.09

Two types of moderator materials, yttrium hydride (YH_2_) and yttrium deuteride (YD_2_), were analyzed for the transmutation rate, support ratio, and power peaking using the moderator volume ratio as a parameter. Power peaking is defined by the ratio of the maximum power pin to the average power pin in the second layer fuel assembly of the outer core.

**Figure 4:**
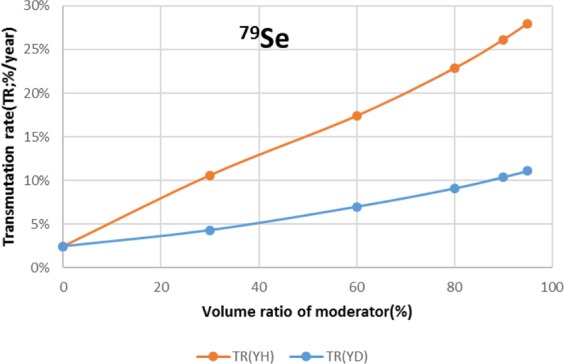
Dependence of transmutation rate of ^79^Se on volume ratio of moderators. As a moderator volume increases, the transmutation rate increases. The transmutation rate using YH_2_ is higher than that using YD_2_.

**Figure 5:**
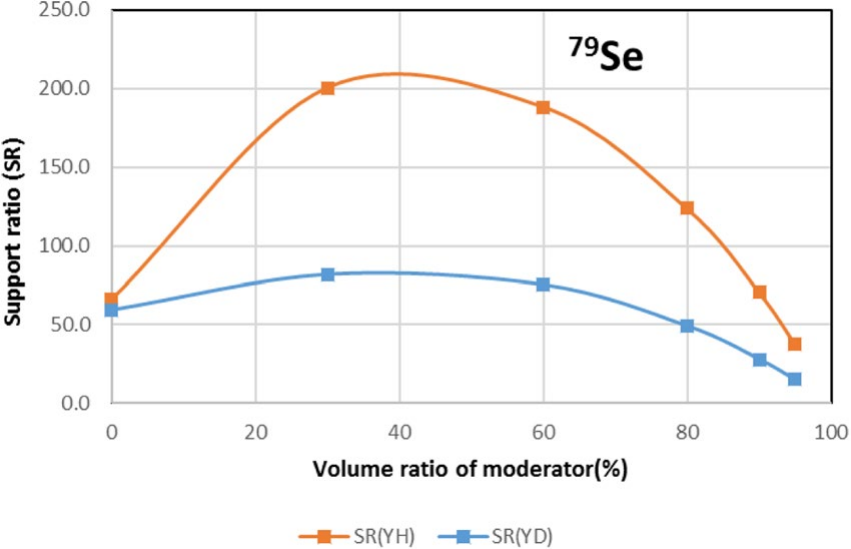
Dependence of support ratio of ^79^Se on volume ratio of moderators. The support ratio has a convex distribution for YH_2_ and becomes maximum at a volume ratio of between 30% and 60%. For YD_2_, the support ratio becomes the maximum at a volume ratio of between 0% and 40%, but the change in the range is small.

**Figure 6:**
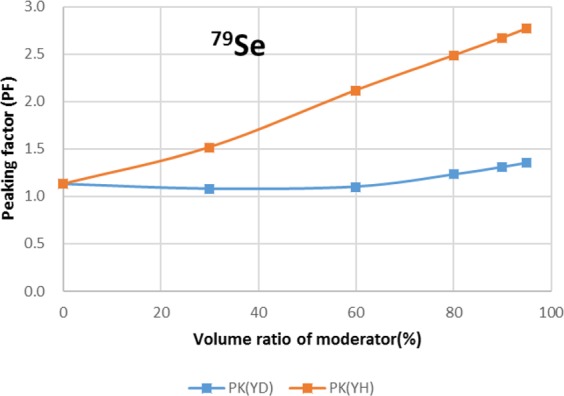
Dependence of power peaking of ^79^Se target assembly on volume ratio of moderators. Power peaking does not change significantly with YD_2_ even when the moderator ratio changes. In the case of YH_2_, power peaking increases greatly with the moderator ratio of YH_2_.

**Figure 7:**
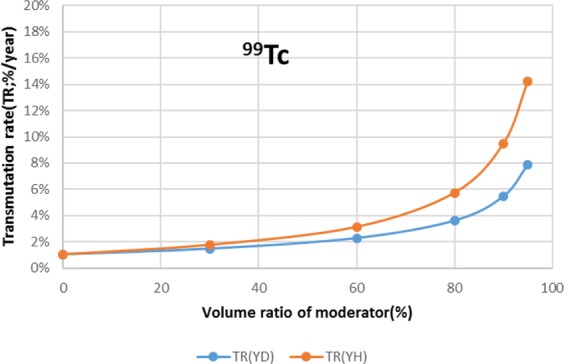
Dependence of transmutation rate of ^99^Tc on volume ratio of moderators. The transmutation rate increases as the moderator volume increases. When the moderator volume ratio exceeds 80%, the transmutation rate increases significantly. The transmutation rate using YH_2_ is higher than that using YD_2_.

**Figure 8:**
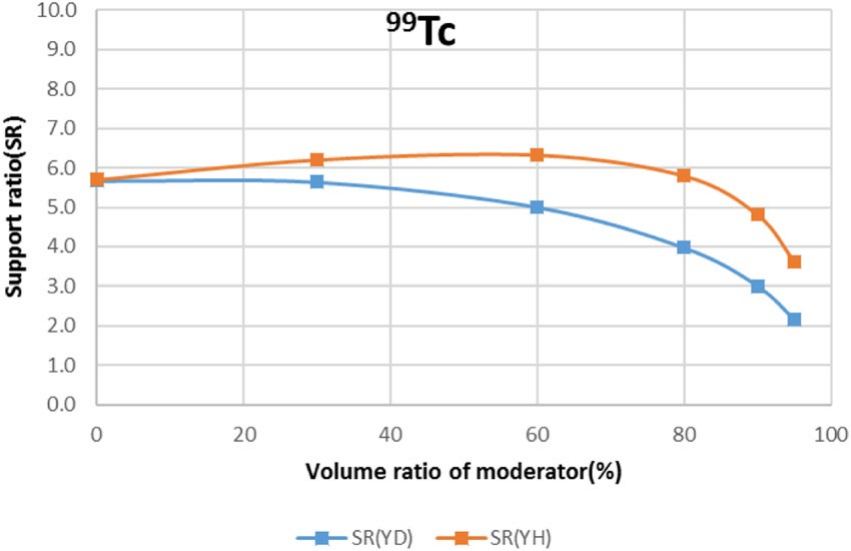
Dependence of support ratio of ^99^Tc on volume ratio of moderators. The support ratio has a convex distribution for YH_2_ and becomes the maximum at a volume ratio of between 30% and 80%. In the case of YD_2_, the support ratio decreases as the volume ratio of the moderator increases.

**Figure 9:**
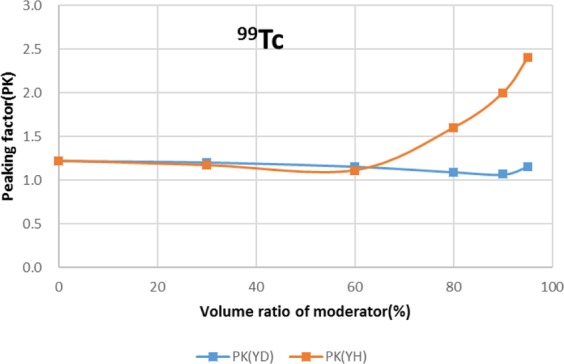
Dependence of power peaking of ^99^Tc target assembly on volume ratio of moderators. In the case of YD_2_, power peaking is almost constant over the entire range of the moderator ratio. In the case of YH_2_, power peaking increases significantly when the volume ratio of YH_2_ exceeds 60%.

**Figure 10:**
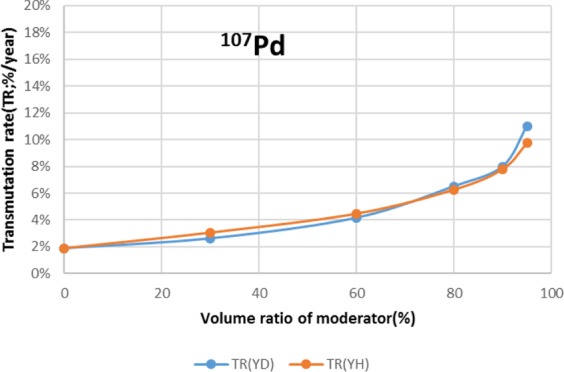
Dependence of transmutation rate of ^107^Pd on volume ratio of moderators. The transmutation rate increases as the moderator volume increases. The transmutation rate using YH_2_ and YD_2_ is almost the same up to 80% of the moderator volume ratio. When the moderator volume ratio is 80% or greater, the transmutation rate of YD_2_ becomes higher than that of YH_2_.

**Figure 11:**
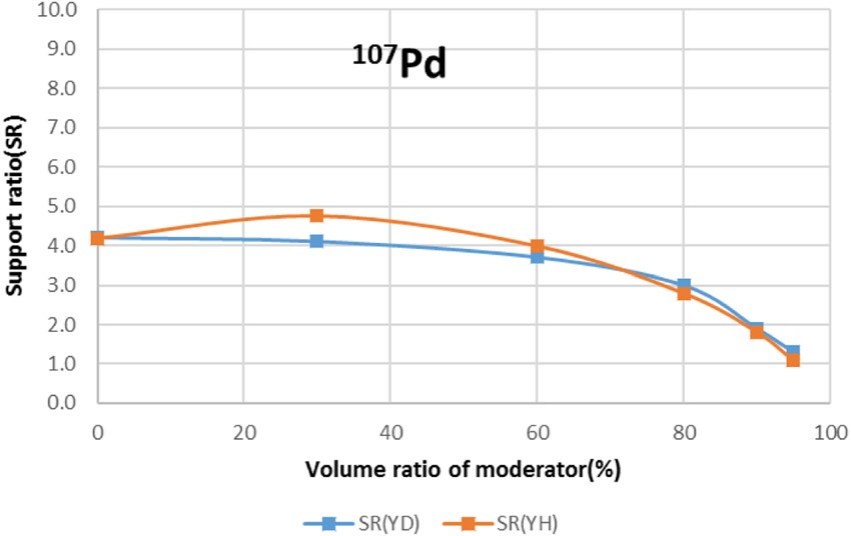
Dependence of support ratio of ^107^Pd on volume ratio of moderators. The support ratio has a convex distribution for YH_2_ and becomes the maximum at a volume ratio of between 20% and 40% volume ratio. In the case of YD_2_, the support ratio decreases as the volume ratio of the moderator increases.

**Figure 12:**
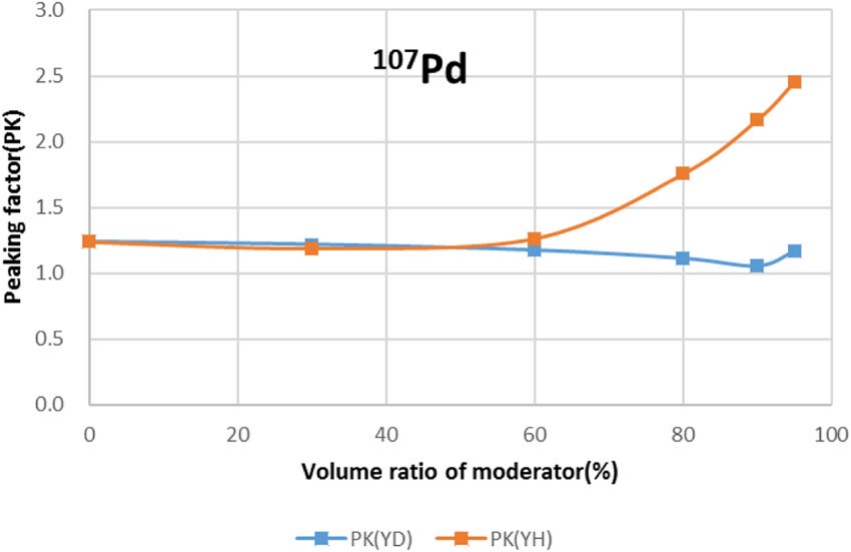
Dependence of power peaking of ^107^Pd target assembly on volume ratio of moderators. In the case of YD_2_, power peaking is almost constant over the entire range of the moderator ratio. In the case of YH_2_, power peaking increases significantly when the volume ratio of YH_2_ exceeds 60%.

**Figure 13:**
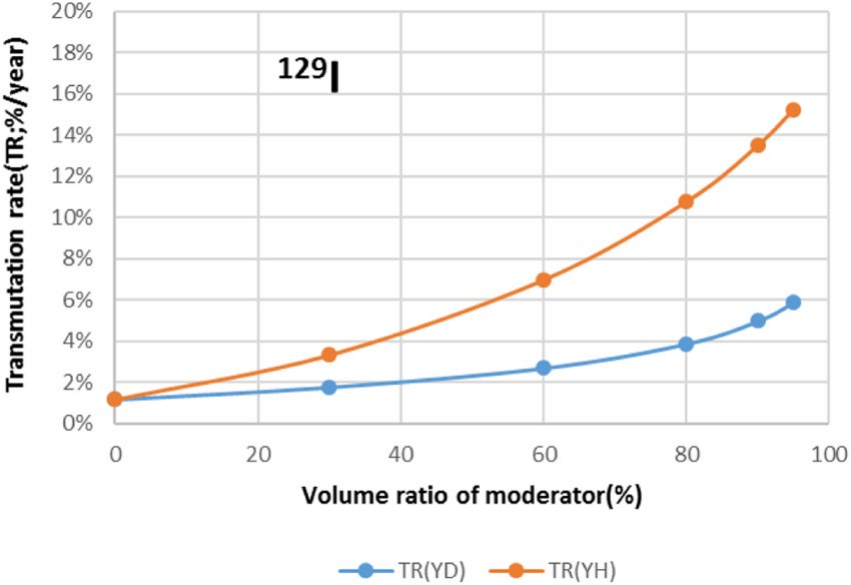
Dependence of transmutation rate of ^129^I on volume ratio of moderators. As the moderator volume ratio increases, both transmutation rates increase. The transmutation rate using YH_2_ is higher than that using YD_2_.

**Figure 14:**
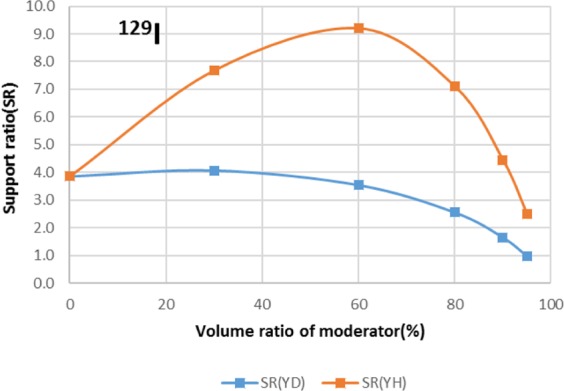
Dependence of support ratio of ^129^I on volume ratio of moderators. The support ratio has a convex distribution for YH_2_ and becomes the maximum at a volume ratio of between 50% and 70%. For YD_2_, the support ratio becomes the maximum at a volume ratio of between 0% and 40%.

**Figure 15:**
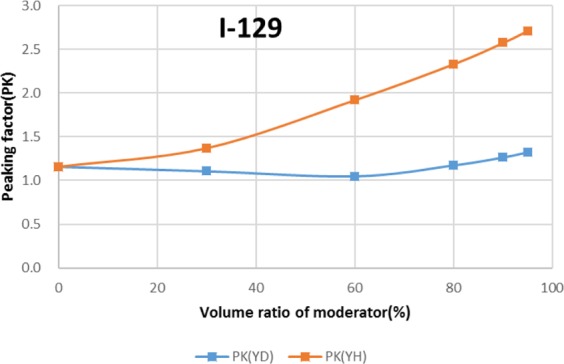
Dependence of power peaking of ^129^I target assembly on volume ratio of moderators. Power peaking does not change significantly with YD_2_ over the entire range of the moderator ratio. In the case of YH_2_, power peaking increases greatly with the moderator ratio of YH_2_.

The results are shown in Figs. 4–15 It has been found that by increasing the moderator volume ratio, a high transmutation rate can be obtained regardless of the moderator material. The transmutation rate using YH_2_ is higher than that using YD_2_. On the other hand, the support ratio was found to have a convex distribution for YH_2_ and becomes the maximum at a volume ratio of between 30% and 60%. For YD_2_, the support ratio became the maximum when the volume ratio was 0% to 40%; however, the change in the range was small. The power peaking did not change significantly with YD_2_ even when the moderator ratio changed. However, in the case of YH_2_, the power peaking increased with the moderator ratio, and it was found that the power peaking tends to rise suddenly from a volume ratio of 80%. The power peaking is approximately 1.25 in the conventional core. Even if the fuel pellets are replaced with high density hollow pellets that allow a higher linear heat rate than the conventional low density solid pellets, power peaking needs to be suppressed to approximately 1.8 due to the maximum linear heat rate limitation.

Based on the results obtained from the parameter survey, under the condition of SR > 1, the moderator material and volume ratio that allow for a high transmutation rate of approximately 8%/year were selected for each nuclide.

For ^79^Se, both YH_2_ and YD_2_ have moderator volume ratios with a transmutation rate of about 8%/year; however, a 90% YD_2_ volume ratio was selected from the viewpoint of power peaking. Under this condition, the support ratio was approximately 28, and SR > 1 was satisfied.

For ^99^Tc, the moderator material and volume ratio of moderator with a transmutation rate of approximately 9.5%/year is YH_2_ and the volume ratio is 90%. Under this condition, the support ratio was approximately 4.8, and SR > 1 was satisfied. On the other hand, power peaking is approximately 2.0; and even if a high-density hollow pellet is adopted, it is considered that the maximum linear heat rate limitation is not satisfied.

For ^107^Pd, the moderator materials at which the transmutation rate was approximately 8%/year were YH_2_ and YD_2_, and the volume ratio was approximately 90%. However, from the viewpoint of power peaking, the 90% volume ratio of YD_2_ was selected. Under this condition, the support ratio was approximately 2, and SR > 1 was satisfied.

For ^129^I, the moderator material with a transmutation rate of about 14%/year was YH_2_, and the volume ratio of the moderator was 90%. Under this condition, the support ratio was approximately 4.3, and SR > 1 was satisfied. On the other hand, power peaking was approximately 2.5, and even if a high-density hollow pellet is adopted, it is considered that the maximum linear heat rate limitation is not satisfied.

For ^99^Tc and ^129^I, it was found that power peaking needs to be suppressed.

When YH_2_ is used as the moderator material, since the slowing down power is high, a large amount of thermal neutrons generated by moderation leaks to the core side. Since the fission cross section of the thermal energy group of fuel nuclides is large, a power peak occurs in the fuel assembly. Therefore, two methods are considered: a method of mixing YH_2_ and YD_2_ with lower slowing down power to control the amount of generated thermal neutrons, and a method of installing thermal neutron absorbing material to prevent the generated thermal neutrons from leaking to the core side.

**Figure 16:**
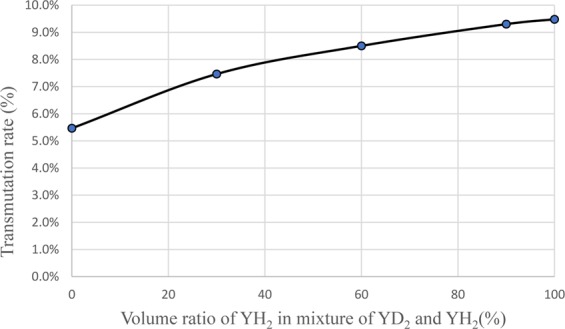
Dependence of transmutation rate for ^99^Tc on volume ratio of YH_2_ in the moderator. The transmutation rate increases as the volume ratio of YH_2_ in the mixed moderator of YH_2_ and YD_2_ increases.

**Figure 17:**
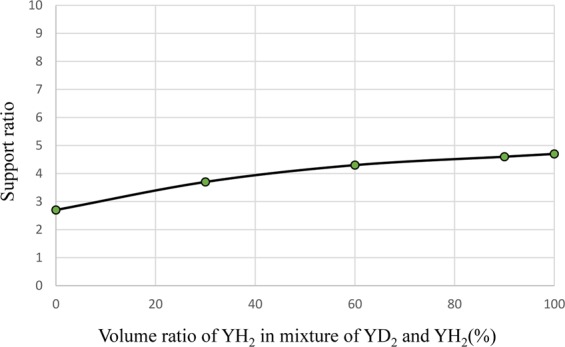
Dependence of support ratio for ^99^Tc on volume ratio of YH_2_ in the moderator. The support ratio increases as the volume ratio of YH_2_ in the mixed moderator of YH_2_ and YD_2_ increases.

**Figure 18:**
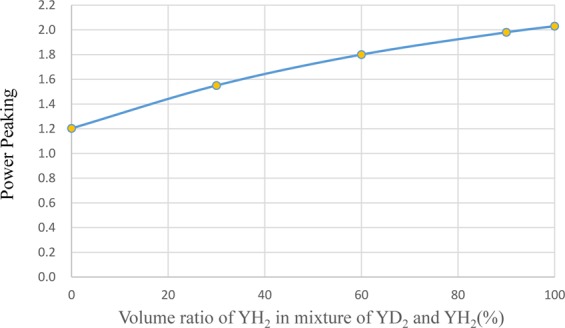
Dependence of power peaking for ^99^Tc target assembly on volume ratio of YH_2_ in the moderator. The power peaking increases as the volume ratio of YH_2_ in the mixed moderator of YH_2_ and YD_2_ increases.

As a method to reduce power peaking of the ^99^Tc target, a measure was taken to mix YH_2_ and YD_2_ as moderator materials. Figure 16 shows the dependence of transmutation rate for ^99^Tc on the volume ratio of YH_2_ in the moderator. Figure 17 shows the dependence of support ratio for ^99^Tc on the volume ratio of YH_2_ in the moderator. Figure 18 shows the dependence of power peaking for ^99^Tc target assembly on the volume ratio of YH_2_ in the moderator. From these figures, the YH_2_ volume ratio in moderator was 60% when power peaking was approximately 1.8, and the transmutation rate was approximately 7.9%/year. Under these conditions, the support ratio was approximately 4.3, and SR > 1 was satisfied.

**Figure 19:**
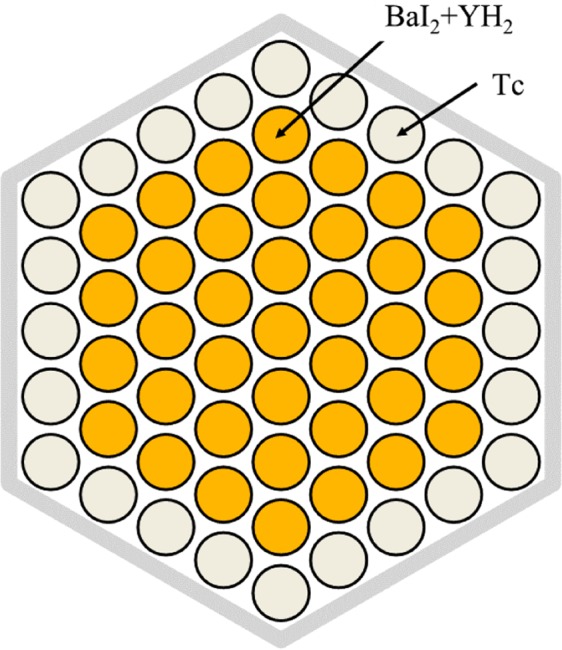
Arrangement of ^129^I pins and ^99^Tc pins in LLFP target assembly. The ^99^Tc target pins replace the ^129^I target pins on the outer first layer in the ^129^I target assembly. The ^99^Tc target pins act as a thermal neutron filter that absorbs thermal neutrons.

**Figure 20:**
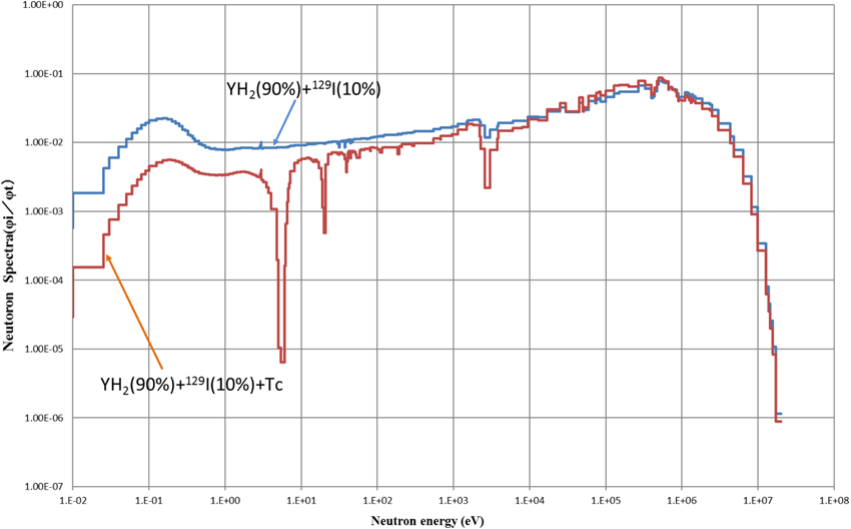
Changes in the neutron spectrum of the outer fuel pin of the adjacent fuel assembly with and without the Tc pin in the LLFP target assembly. When ^99^Tc is installed, the neutron flux in the thermal neutron region decreases. This is effective in suppressing the power of the fuel pins near the LLFP target assembly.

**Figure 21:**
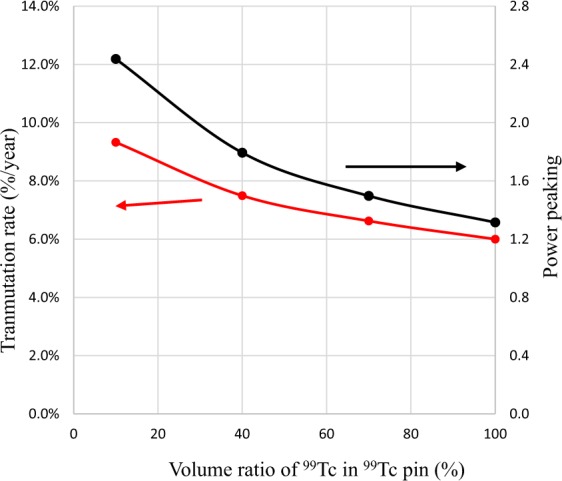
Dependence of power peaking and transmutation rate on volume ratio of ^99^Tc in ^99^Tc pin. As the volume ratio of ^99^Tc in the Tc pin increases, ^129^I transmutation rate and power peaking in the assembly decrease. When the ^99^Tc volume ratio was approximately 40%, power peaking was approximately 1.8, and the transmutation rate at that time was 7.5%/year.

To reduce power peaking in the ^129^I target, a method of installing a thermal neutron absorber was adopted because the transmutation rate of ^129^I was reduced when using the method of mixing YH_2_ and YD_2_. Many neutron absorbing materials are conceivable as thermal neutron absorbing material. As a new method, we examined Tc, which is also the target of LLFP transmutation. A new idea to replace the ^129^I target pins on the outer first layer of the ^129^I target assembly with the ^99^Tc target pins was studied. Figure 19 shows the arrangement of ^129^I pins and ^99^Tc pins in ^129^I target assembly. Figure 20 shows the neutron spectrum with and without the ^99^Tc. When ^99^Tc is installed, the neutron flux in the thermal neutron region decreases. Its effectiveness in suppressing the power of the fuel pins near the LLFP target assembly can be seen. Figure 21 shows the analysis results of transmutation rate and power peaking when the ^99^Tc volume of the ^99^Tc pin in the outer first layer of the ^129^I target assembly is changed. When the ^99^Tc volume ratio was about 40%, power peaking was approximately 1.8, and the transmutation rate at that time was 7.5%/year. In this case, the support ratio was 1.5 or more, and SR > 1 was satisfied.

Table 3 summarizes the analysis results of transmutation target assemblies with high transmutation characteristics when LLFP is loaded in a fast reactor. It was found that all four nuclides can achieve high transmutation rates while keeping the limiting conditions for power peaking.

**Table 3:** Transmutation performance of each LLFP.

LLFP nuclides	Amount of loaded LLFP (g)	Amount of transmutation (g/year)	Transmutation rate (%/year)	SR	Moderator composition
^79^Se	1.05 × 10^+4^	1.09 × 10^+3^	10.4	28.2	YD_2_
^99^Tc	2.98 × 10^+5^	2.34 × 10^+4^	7.9	4.3	60%YH_2_ + 40%YD_2_
^107^Pd	6.58 × 10^+4^	5.26 × 10^+3^	8.0	1.8	YD_2_
^129^I	3.27 × 10^+4^	2.45 × 10^+3^	7.5	1.5	YH_2_+ (Tc)

The pin temperature and pin pressure loaded with LLFP were evaluated. As a result, all pin temperatures were below the limit value. Also, the pin pressure of the BaI_2_ loaded pin, which generates Xe gas, was approximately 50 kg/cm^2^ after 3 years in the reactor. It was found that the cumulative damage factor of the cladding tube due to the inner pressure of the cladding tube was well below the limit value 1. The integrity of the LLFP target pin was confirmed. The cladding tube temperature limit was set at 600 °C in consideration of the melting point except for Se. The Se loading form was ZnSe, and the pellet center temperature was required to be 500 °C or less in order to prevent sublimation. Therefore, the cladding tube temperature for ZnSe was set at 470 °C. The required coolant flow rate was 1.35 kg/s for all LLFP assemblies.

### Study on material properties of moderator and LLFP

#### Compatibility of cladding material and LLFP

Among the target candidates, excluding iodine and technetium, whose compatibility with cladding materials has been clarified by previous studies^35,36^, compatibility experiments were conducted on ZnSe and Pd.

From structural observation and elemental analysis of SUS316 steel, no reaction with ZnSe was observed; and it was revealed that ZnSe has good compatibility with SUS316 steel. Regarding Pd and SUS316 steel, the weight changes of SUS316 steel were −0.0001 g and −0.00002 g, both showing weight reduction. From the structural observation and elemental analysis of SUS316 steel, oxide was formed in the surface layer. This is an oxide of Fe, and Pd was not detected. Oxygen is considered to be an impurity contained in the experimental atmosphere; however, it was not observed in the compatibility experiment of ZnSe and SUS316 steel conducted under the same conditions. Therefore, Pd is considered to influence this oxide formation. For this reason, when loading a Pd target, it is necessary to pay attention to the management of the atmosphere in the cladding tube.

#### Experiments on fabrication and retention of Y hydride and Y deuteride

**Figure 22:**
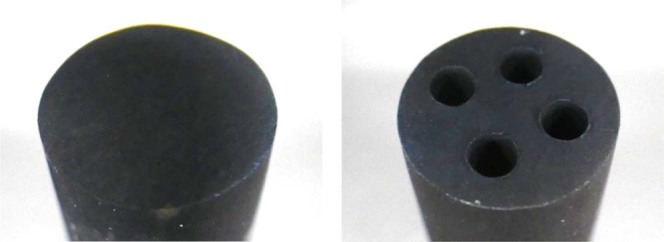
Fabrication results of YD_2_ pellets. YD_2_ moderator pellets can be manufactured firmly, and the holes for LLFP can also be manufactured securely.

**Figure 23:**
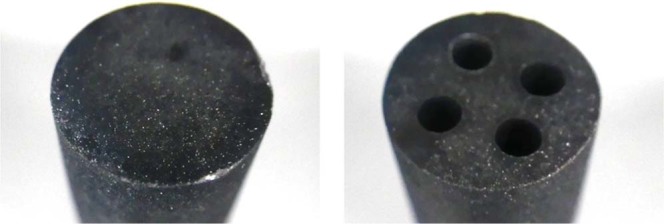
Fabrication results of YH_2_ pellets. YH_2_ moderator pellets can be manufactured firmly, and the holes for LLFP can also be manufactured securely.

Y hydrides and YD_2_ used as neutron moderators were fabricated in pellets; and they were tested for the evaluation of retention characteristics of hydrogen and deuterium under temperature conditions of reactor operation. Figure 22 shows the fabrication results of YD_2_ pellets. Figure 23 shows the fabrication results of YH_2_ pellets. It was found that both YD_2_ and YH_2_ moderator pellets can be fabricated securely.

As for YH_2_ and YD_2_, it was found that when YH_3_ or YD_3_ is produced, the shape stability of targets is remarkably inferior, and the amount of dissociation of hydrogen or deuterium increases at low temperatures. A fabrication method that does not produce YH_3_ or YD_3_ was adopted.

As a result of evaluating the release amount of hydrogen or deuterium at high temperatures of Zr hydrides and deuterides and Y hydrides and deuterides by the thermal desorption gas analysis (TDS), ZrH_2_ and ZrD_2_ released almost all hydrogen or deuterium up to 600 °C. In YH_2_ and YD_2_, the amount of hydrogen or deuterium released up to 600 °C. was small. Therefore, it was clarified that Y is superior to Zr in hydrogen retention performance up to 600 °C, which is assumed to be the reactor temperature.

## Conclusions

The purpose of this study was to clarify the method to achieve high transmutation rates of four long-lived fission products (^79^Se, ^99^Tc, ^107^Pd, and ^129^I) using a fast reactor.

For ^79^Se, from the viewpoint of high transmutation rate and reduction of power peaking of adjacent fuel assemblies, the volume ratio of ZnSe and YD_2_ was set at 1:9, resulting in a transmutation rate of 10.4%/year. It was also found that SR achieved approximately 28.

For ^99^Tc, a measure was taken to mix YH_2_ and YD_2_ as the moderator material in order to suppress power peaking of the adjacent fuel assembly. By changing the volume ratio of YH_2_ and YD_2_ to 6:4 and the volume ratio of ^99^Tc and moderator (YH_2_ + YD_2_) to 1:9, the transmutation rate was 7.9%/year. SR was 4.3, satisfying SR > 1.

For ^107^Pd, the transmutation rate was 8.0%/year by setting the volume ratio of Pd and YD_2_ at 1:9 from the viewpoint of high transmutation rate and reduction of power peaking of adjacent fuel assemblies. SR was also found to achieve 1.8.

For ^129^I, a new ^129^I target assembly with a thermal neutron filter was invented to suppress power peaking of adjacent fuel assemblies. As the thermal neutron filter, the outer layer of the ^129^I target assembly was replaced with Tc. By changing the volume ratio of Tc in the Tc pin to 40% and the volume ratio of BaI_2_ to YH_2_ to 1:9, the transmutation rate was 7.5%/year. The SR was 1.5.

The feasibility of the LLFP transmutation target was clarified through experiments on the material properties and fabrication of LLFP targets, YH_2_ and YD_2_ moderators.

The impact of separation characteristics and transmutation rate on ^93^Zr and ^135^Cs will be studied in future.

## Method

### Selection of LLFP target

The selection of materials for the four nuclides was decided under the following considerations.

Se is a metalloid element having a melting point of 221 °C and a boiling point of 685 °C, and is in a liquid phase at the operating temperature of the fast reactor. Therefore, ZnSe was selected as a compound form t that is a solid phase when loaded into the reactor core. ZnSe has a melting point of 1526 °C, and thermal analysis results confirm its stability up to 500 °C. Since the transmutation target improves transmutation efficiency by mixing with the neutron moderator, the transmutation target of ZeSe is a mixed complex with Yttrium deuteride.

Tc is a transition metal having a melting point of 2157 °C and a boiling point of 4265 °C, and even a Tc metal can maintain a solid state in a fast reactor. However, the transmutation target improves the transmutation efficiency by mixing with a neutron moderator. Therefore, the transmutation target of Tc is a complex with YH_2_ or/and YD_2_.

Pd is a transition metal having a melting point of 1555 °C and a boiling point of 2964 °C, and even a Pd metal can maintain a solid state in a fast reactor. However, because the transmutation target improves the transmutation efficiency by combining with a neutron moderator, the transmutation target of Pd is a complex with YD_2_.

Iodine is a halogen element having a melting point of 114 °C and a boiling point of 185 °C, and it is in a gas phase at the operating temperature (about 500 °C) of the fast reactor. For this reason, BaI_2_ was selected as a compound form that becomes a solid phase when loaded into the reactor core. BaI_2_ has a melting point of 711 °C. The transmutation ^129^I target is a complex with YH_2_.

In previous study^35^, increasing the ratio of moderator has made the LLFP target hard to sinter when compounded LLFP and moderator material are mixed.

Therefore, the transmutation target of I or Se is a complex in which BaI_2_ or ZnSe compound is inserted into the holes provided in the moderator pellet.

The transmutation target of ^99^Tc or Pd is a complex in which ^99^Tc or Pd wire is inserted into the small holes provided in the moderator pellets.

### Reactor physics analysis

Using a 300 MWe class sodium cooled, MOX fueled fast reactor, aiming at a high transmutation rate for four nuclides of ^79^Se, ^99^Tc, ^107^Pd and ^129^I, the transmutation rate, support ratio, and power peaking coefficient were analyzed using the following parameters: type of moderator (deuteride, hydride), ratio of LLFP and moderator in the LLFP target, and the target arrangement in a LLFP target assembly. Analysis was performed using the MVP code^37^ and MVP-burn code^38^. The cross section library used was JENDL-4.0^39^ installed in the MVP code. The MVP 3D full reactor model included accurate representation of the configuration of the assembly and pin. The MVP calculations were performed under the same conditions with the previous paper^27^. The uncertainties of the present calculation were almost same with the previous results^27^. For the four nuclides of ^79^Se, ^99^Tc, ^107^Pd, and ^129^I, the target assemblies were loaded on the first layer of the blanket. The structure and material of the target assembly are the same as those of the blanket fuel assembly.

### Material property experiments

For evaluating the feasibility of LLFP transmutation, it is important to confirm material compatibility. The following experiments were conducted in relation to LLFP target materials, hydride and deuteride characteristics, LLFP target production, LLFP separation and recovery, etc. They are experiments for the evaluation of coexistence with cladding materials and LLFP, experiments for LLFP production methods, experiments for production and retention performance of hydride and deuteride, and experiments for separation and recovery methods of irradiated LLFP.

#### Compatibility experiments with cladding materials

In the compatibility experiment, SUS316 steel selected as a cladding material in a quartz tube was brought into contact with each LLFP candidate compound powder, heated to a high temperature, and held for 500 hours. The heating temperature was set at 500 °C from the viewpoint of the stability of ZnSe and at 650 °C for Pd.

No experiments for iodine and Tc were carried out because the stability of these elements was confirmed by previous experimental results^35,36^.

#### Fabrication method of LLFP targets

There are several fabrication methods for ZnSe, which is a candidate form of ^79^Se, including a fabrication of a single crystal material by vapor phase method^40^; however, the fabrication method using vapor phase has high LLFP diffusivity. There is concern about an increase in the loss rate in the fabrication process. Therefore, synthesis by thermal decomposition of metal alkoxides that has been studied is considered suitable as a fabrication method for handling radioactive ^79^Se because there is no generation of a gas phase during fabrication and the temperature during fabrication is low^41^.

For BaI_2_, which is a candidate form of I, a method of synthesizing BaI_2_ by using red phosphorus to change I to IH and reacting with a barium compound such as barium carbonate (BaCO_3_) is effective. However, BaI_2_ is highly hygroscopic and easily forms hydrates, and there is concern about BaI_2_ hydrates promoting corrosion of stainless steel, which is a cladding material. It is necessary to perform sufficient dehydration treatment at the final stage in the production of the above.

Since Tc and Pd are in a single form, the fabrication method of these LLFPs is to increase the purity of the recovered product from the reprocessing process. Tc is assumed to be contained in the nitric acid solution in the reprocessing dissolution process, and Tc can be purified and recovered with high efficiency by denitration with formic acid. Pd is present in the insoluble residue and solution in the fuel dissolution process, and it can be purified by separation with the aqua regia dissolved zinc or formate after separation of the insoluble residue. Thus, these techniques can be applied to the purification of Tc and Pd.

#### Experiments of fabrication and retention of hydrides and deuterides

In order to clarify the retention characteristics of hydrogen in YH_2_ and deuterium in YD_2_, TDS and high temperature X-ray diffraction (High temperature XRD) using the fabricated YH_2_ and YD_2_ was performed.

## References

[1] Gray WJ (1982). Fission product transmutation effects on high-level radioactive waste forms. Nature.

[2] Ramspott, L. D. *et al*. *Impacts of new developments in partitioning and transmutation on the disposal of high*-*level nuclear waste in a mined geologic repository*. (1992).

[3] Salvatores M, Slessarev I, Uematsu M (1994). A global physics approach to transmutation of radioactive nuclei. Nuclear Science and Engineering.

[4] Walker C, Nicolaou G (1995). Transmutation of neptunium and americium in a fast neutron flux: EPMA results and KORIGEN predictions for the superfact fuels. Journal of Nuclear Materials.

[5] Kloosterman, J. L. & Li, J. Transmutation of Tc-99 and I-129 in fission reactors. A calculational study. In *Netherlands Energy Research Foundation* (*ECN*), *Petten* (*Netherlands*). *Funding organisation: Commission of the European Communities*, *Brussels* (*Belgium*) (1995).

[6] Tommasi J, Delpech M, Grouiller J-P, Zaetta A (1995). Long-lived waste transmutation in reactors. Nuclear Technology.

[7] Salvatores M, Slessarev I, Uematsu M, Tchistiakov A (1998). The neutronic potential of nuclear power for long-term radioactivity risk reduction. Progress in Nuclear Energy.

[8] Wakabayashi T, Higano N (1998). Study on MA and FP transmutation in fast reactors. Progress in Nuclear Energy.

[9] OECD-NEA. *Actinide and fission product partitioning and transmutation Status and assessment report*. (1999).

[10] Hwang I (2000). The concept of proliferation-resistant, environment-friendly, accident-tolerant, continual and economical reactor (PEACER). Progress in Nuclear Energy.

[11] Messaoudi N, Tommasi J (2002). Fast burner reactor devoted to minor actinide incineration. Nuclear Technology.

[12] Wakabayashi T (2002). Transmutation characteristics of MA and LLFP in a fast reactor. Progress in Nuclear Energy.

[13] Suzuki M, Ezoubtchenko A, Akatsuka H, Matsuura H, Takagi R (2002). Isotope separation required in SCNES and future subjects. Progress in Nuclear Energy.

[14] Takaki, N. & Mizuno, T. Design study on sodium-cooled fast reactor core loaded with LLFP transmutation sub-assemblies. *In Proceedings of GENES4/ANP2003* (2003).

[15] Salvatores M (2005). Nuclear fuel cycle strategies including partitioning and transmutation. Nucl. Eng. Des..

[16] Aoyama T, Maeda S, Maeda Y, Suzuki S (2005). Transmutation of technetium in the experimental fast reactor JOYO. Journal of Nuclear and Radiochemical Sciences.

[17] OECD-NEA. *Advanced Nuclear Fuel Cycles and Radioactive Waste Management*. (2006).

[18] Warin, D. Status of the french research on partitioning and transmutation. In *MRS Proceedings*. (2006).

[19] OECD-NEA. *Physics and Safety of Transmutation Systems*, *A Status Report*. (2006).

[20] OECD-NEA. *Regulating the long*-*term safety of geological disposal Towards a common understanding of the main objectives and bases of safety criteria*. (2007).

[21] Osaka M (2007). Research and development of minor actinide-containing fuel and target in a future integrated closed cycle system. Journal of Nuclear Science and Technology.

[22] Yokoyama, T., Wakabayashi, T., Tachi, Y., Takaki, N. & Matsuyama, S. New target concepts for increase in transmutation rate of LLFPs in FBR recycle system. In *Proceedings of International Conference on the Nuclear Fuel Cycle*. (2009).

[23] Tachi, Y., Wakabayashi, T. & Yokoyama, T. Study on target fabrication for LLFP transmutation by fast reactors. *In**Proceedings of Global* 2009, Paris (2009).

[24] OECD-NEA. *Potential Benefits and Impacts of Advanced Nuclear Fuel Cycles with Actinide Partitioning and Transmutation*. (2011).

[25] Gonzalez-Romero E (2011). Impact of partitioning and transmutation on the high level waste management. Nuclear Engineering and Design.

[26] Liu B (2014). Transmutation of minor actinides in the pressurized water reactors. Annals of Nuclear Energy.

[27] Chiba, S. *et al*. Method to Reduce Long-lived Fission Products by Nuclear Transmutations with Fast Spectrum Reactors. *Scientific Reports* **7**, 13961, 10.1038/s41598-017-14319-7. 10.1038/s41598-017-14319-7 PMC5654822 29066843

[28] Wakabayashi T (2019). Core concept of simultaneous transmutation of six LLFP nuclides using a fast reactor. Nuclear Engineering and Design.

[29] Sasaki, Y. *et al*. Extraction and Separation of Se, Zr, Pd, and Cs Including Long-lived Radionuclides, *Solvent Extraction Research and Development*, Japan, Vol24, No2, 113–122 (2017).

[30] Soelberg Nick R., Garn Troy G., Greenhalgh Mitchell R., Law Jack D., Jubin Robert, Strachan Denis M., Thallapally Praveen K. (2013). Radioactive Iodine and Krypton Control for Nuclear Fuel Reprocessing Facilities. Science and Technology of Nuclear Installations.

[31] Volckaert, G. & Mallants, D. Long-Term Environmental Impact of Underground Disposal of P&Twaste, https://www.oecd-nea.org/pt/docs/iem/mol98/session6/SVIpaper1.pdf.

[32] Vetrano J (1971). Hydrides as neutron moderator and reflector materials. Nuclear Engineering and Design.

[33] Yokoyama, T., Wakabayashi, T., Tachi, Y. & Nagata, A. Optimizing pin layout in transmutation rate of long-life FP with deuteride moderator for fast reactors. In *Proceedings of GLOBAL 2011*, *Makuhari*, *Japan* (2011).

[34] Wakabayashi Toshio (2013). Improvement of Core Performance by Introduction of Moderators in a Blanket Region of Fast Reactors. Science and Technology of Nuclear Installations.

[35] Tachi, Y. & Wakabayashi, T. Fabrication of BaI_2_-ZrH_2_-x Composite for I-129 Transmutation Target, Proceedings of GLOBAL2011, Makuhari, Japan, Dec. 11–16 (2011).

[36] Konings RJM, Stalios AD, Walker CT, Cocuaud N (1998). Transmutation of technetium: results of the EFTTRA-T1 experiment. J. Nuclear Materials.

[37] Mori, T. & Nakagawa, M. Mvp/gmvp: general purpose Monte Carlo codes for neutron and photon transport calculations based on continuous energy and multigroup methods. *JAERI Data/Code*, JAERI-Data/Code–94–007 (1994).

[38] Okumura K, Mori T, Nakagawa M, Kaneko K (2000). Validation of a continuous-energy Monte Carlo burn-up code MVP-BURN and its application to analysis of post irradiation experiment. Journal of Nuclear Science and Technology.

[39] Shibata K (2011). JENDL-4.0: a new library for nuclear science and engineering. Journal of Nuclear Science and Technology.

[40] Korostelin YY, Kozlovsky VI, Nasibov AS, Shapkin PV (1996). Vapour growth and characterization of bulk ZnSe single crystals. J. Crystal Growth.

[41] Baba N, Hattori S, Kawasaki K, Ozaki Y (1997). Preparation of ZnSe by Thermal Decomposition of Metal Alkoxide. Journal of the Ceramic Society of Japan.

